# Natural selection on *HFE* in Asian populations contributes to enhanced non-heme iron absorption

**DOI:** 10.1186/s12863-015-0223-y

**Published:** 2015-06-10

**Authors:** Kaixiong Ye, Chang Cao, Xu Lin, Kimberly O O’Brien, Zhenglong Gu

**Affiliations:** Division of Nutritional Sciences, Cornell University, Ithaca, NY USA; Key Laboratory of Nutrition and Metabolism, Institute for Nutritional Sciences, Shanghai Institutes for Biological Sciences, Chinese Academy of Sciences and Graduate University of the Chinese Academy of Sciences, Shanghai, China

**Keywords:** Adaptation, Iron Homeostasis, Absorption, *HFE*

## Abstract

**Background:**

*HFE*, a major regulator of iron (Fe) homeostasis, has been suggested to be under positive selection in both European and Asian populations. While the genetic variant under selection in Europeans (a non-synonymous mutation, C282Y) has been relatively well-studied, the adaptive variant in Asians and its functional consequences are still unknown. Identifying the adaptive *HFE* variants in Asians will not only elucidate the evolutionary history and the genetic basis of population difference in Fe status, but also assist the future practice of genome-informed dietary recommendation.

**Results:**

Using data from the International HapMap Project, we confirmed the signatures of positive selection on *HFE* in Asian populations and identified a candidate adaptive haplotype that is common in Asians (52.35–54.71 %) but rare in Europeans (5.98 %) and Africans (4.35 %). The T allele at tag SNP rs9366637 (C/T) captured 95.8 % of this Asian-common haplotype. A significantly reduced *HFE* expression was observed in individuals carrying T/T at rs9366637 compared to C/C and C/T, indicating a possible role of gene regulation in adaptation. We recruited 57 women of Asian descent and measured Fe absorption using stable isotopes in those homozygous at rs9366637. We observed a 22 % higher absorption in women homozygous for the Asian-common haplotype (T/T) compared to the control genotype (C/C). Additionally, compared with a group of age-matched Caucasian women, Asian women exhibited significantly elevated Fe absorption.

**Conclusions:**

Our results indicate parallel adaptation of *HFE* gene in Europeans and Asians with different genetic variants. Moreover, natural selection on *HFE* may have contributed to elevated Fe absorption in Asians. This study regarding population differences in Fe homeostasis has significant medical impact as high Fe level has been linked to an increased disease risk of metabolic syndromes.

**Electronic supplementary material:**

The online version of this article (doi:10.1186/s12863-015-0223-y) contains supplementary material, which is available to authorized users.

## Background

Adaptations to dietary intake during human evolution have shaped the human genome and resulted in ethnic variability in nutrient utilization and risk of diseases [1–3]. Classic examples have been established for the prevalence of lactase persistence in Northern Europeans and East Africans as an adaptation to milk consumption [4], high copy number of *AMY1* in populations ingesting starch-rich diets [5], and the Asian alcohol flush reaction which evolved as an adaptive response to alcohol consumption after rice domestication [6]. The recent advent of high-throughput genotyping and sequencing technology enables genome-wide scans for signals of positive selection and generates many hypotheses that await functional testing and confirmation [7, 8]. Establishing these adaptive variants is clinically relevant because the incompatibility between genetic adaptations in the past and the modern dietary environment could underlie many metabolic diseases in the current society [1–3].

Iron (Fe) is an essential micronutrient involved in oxygen transport, oxidative metabolism and immune function [9, 10]. Iron deficiency (ID) is one of the most widespread micronutrient deficiencies worldwide and may lead to ID anemia, causing chronic fatigue, reduced work productivity, impaired immune response, poor pregnancy outcome, and delayed physical and cognitive development in infants [11–14]. On the other hand, Fe overload is also detrimental because of its participation in redox reaction, generating free radicals [15]. Fe overload is implicated in a number of common chronic diseases, including type II diabetes, cirrhosis, liver fibrosis, cardiomyopathy and cancer [11, 16–22]. Maintaining Fe homeostasis is fundamentally critical for human health. Interestingly, there is no mechanism for regulated excretion of Fe and Fe homeostasis relies primarily on the tight regulation of non-heme Fe absorption from the diet [15]. Dietary Fe comes in two forms: heme Fe (animal-based products) and non-heme Fe (animal- and plant-based products). Heme Fe constitutes only about 10 % of total dietary Fe content in a typical Western diet but accounts for about 2/3 of absorbed iron because of its 4–8 times higher bioavailability than non-heme Fe [15, 23, 24]. For individuals (e.g. vegetarians) or populations consuming predominantly plant-based diets and relying solely on the non-heme form of Fe, they have a much higher risk for ID [24]. In certain Asian populations (e.g. Chinese) with a long tradition of consuming plant-based, iron-poor diets [24–26], genetic variations enhancing non-heme Fe absorption could have been especially beneficial and subject to positive natural selection. However, no such genetic variations have been revealed to date. Furthermore, while population differences in Fe status and the prevalence of ID have been described [12, 27], the potential role of genetic variation underlying these differences has remained largely uncharacterized. In the modern Fe-replete dietary environment, identifying genetic variation enhancing Fe absorption is especially important for future prevention of Fe overload and its associated disorders.

*HFE* is one of the major regulators of non-heme Fe absorption and Fe homeostasis. The gene was the first found to be implicated in hereditary hemochromatosis (HH), an autosomal recessive disorder of Fe metabolism causing excess body Fe accumulation [28, 29]. A non-synonymous mutation of *HFE*, C282Y (rs1800562), causes enhanced non-heme Fe absorption, which could not be appropriately down-regulated even in face of elevated Fe stores. This mutation is responsible for more than 80 % of HH found in Europe [28]. While this mutation has a frequency of 5–14 % in northern European populations, it is nearly absent outside of Europe [30, 31]. The relatively high frequency of this mutation in European populations has been suggested to be a result of recent positive selection, although the underlying selection pressure has been controversial [31–34]. While the hypothesis of dietary adaptation to increase Fe absorption in heterozygous carriers of the mutation has gathered lots of interest, it was shown that C282Y-heterozygous individuals actually do not have elevated dietary Fe absorption [35, 36]. The adaptive effect of this deleterious mutation might lie in the possibility that C282Y homozygotes have Fe-deplete macrophages and therefore gain resistance to macrophage-dwelling pathogens, which could not live without Fe [33, 34]. The sequence variation and haplotype structure at *HFE* are quite different among continental populations, and interestingly, Asian populations possess a high-frequency haplotype, referred to as the Asian-common haplotype, that is rarely observed among European or African populations [37]. This haplotype may have been driven to high frequency by positive selection if it provided a selective advantage. Consistently, a signal of positive selection on *HFE* has been suggested in Chinese populations based on patterns of single nucleotide polymorphism (SNP) allele frequency around the *HFE* gene [38]. However, the possibility of local adaptation of *HFE* in Asia requires further confirmation and the underlying adaptive variants need to be revealed.

We hypothesize that the Asian-common haplotype present at a high frequency in Asian populations is associated with improved Fe stores and has a functional impact on absorption of non-heme Fe. To test this hypothesis, we performed multiple evolutionary analyses and unraveled a regulatory variant in *HFE* with adaptive signals in Asian populations. The impact of this regulatory variant on Fe status and absorption was explored in a group of young Asian women. With the Fe absorption data from this study, we were able to further test for the first time if there is population difference in Fe absorption between Asian and Caucasian women.

## Results

### An Asian-common *HFE* haplotype has positive selection signal and is associated with lower *HFE* expression

**Fig. 1:**
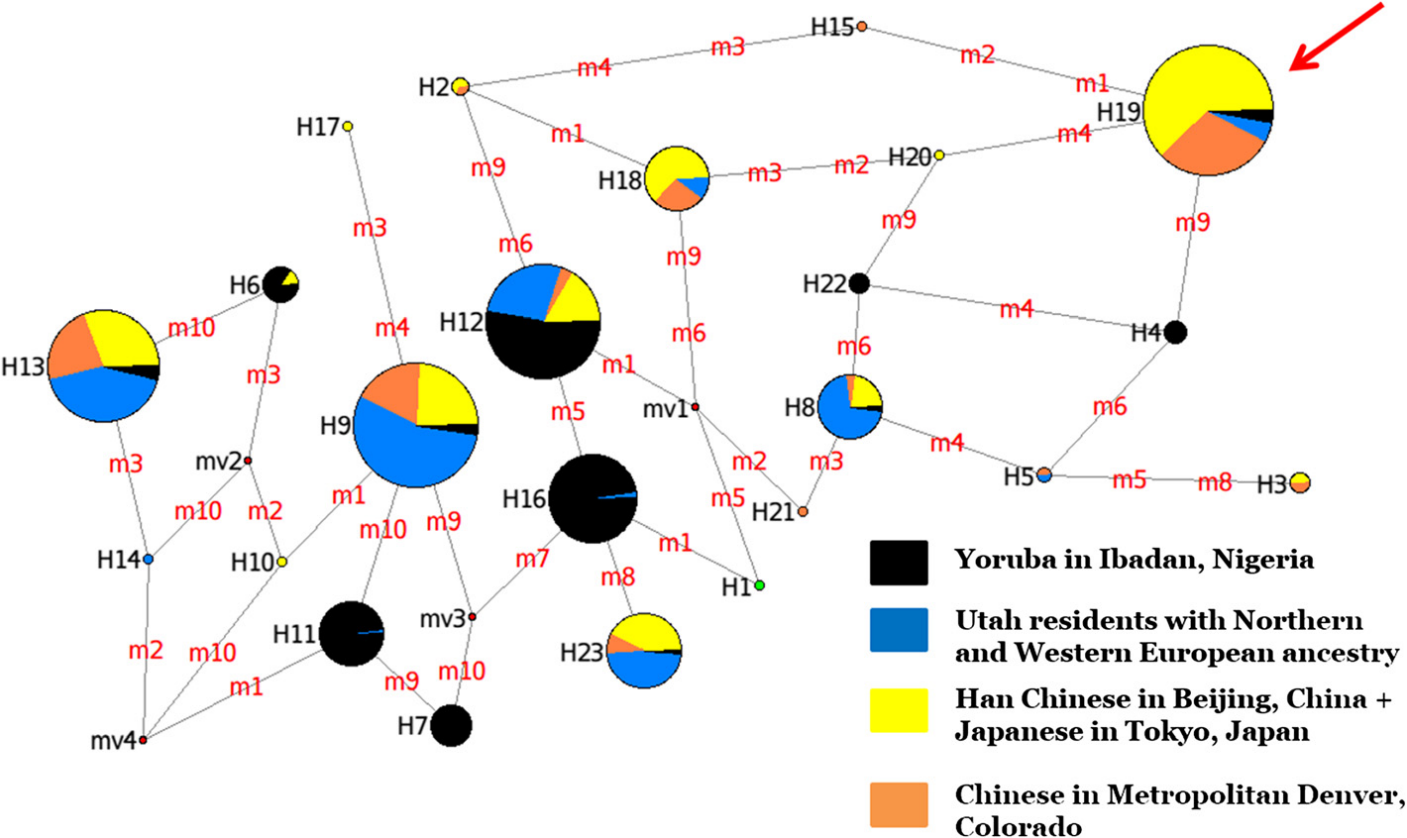
The haplotype structure of *HFE*. SNPs within 20 Kb of *HFE* were used in this analysis. Data were retrieved from Hapmap 3. Each node represents one haplotype and the size of each node is proportional to the frequency of the haplotype among all haplotypes observed. Each node is also a pie chart and each sector represent the contribution of each population. The line between two nodes represents the evolutionary connection between the two haplotypes and the mutations to convert the two haplotypes are indicated on the line. The red arrow points to the Asia-prevalent haplotype, H19. The green node represents the haplotype (H1) observed in Chimpanzees

Haplotype analysis on *HFE* in 487 HapMap individuals from three continents revealed 22 haplotypes (Fig. 1, Additional file 1: Table S1). One haplotype was identified with frequencies of 52.35–54.71 % in East Asian samples, 5.98 % in the North Europeans and 4.35 % in the West Africans. This Asian-common haplotype could be tagged by a single SNP, rs9366637 (ancestral allele C, derived allele T). Among haplotypes carrying T at rs9366637 in all samples, 95.80 % were the Asian-common haplotype (Additional file 1: Table S1). Evolutionary analysis using the iHS method revealed a signal of positive selection on *HFE* only among the Asian samples but not in the European or African samples (empirical *p* value = 0.011, Additional file 1: Figure S1). Specifically, the tag SNP, rs9366637, has an extreme iHS value of -2.89, suggesting that its derived allele, T, is on an unusually long haplotype, probably as a result of positive selection. Consistently, the frequency of T is high in Asian populations but low in Africans and Europeans (Additional file 1: Figure S2). The population frequency divergence is also reflected by the F_ST_ statistic of rs9366637. Its F_ST_ value is 0.23 between Asian and European populations, which is more extreme than the 5 % genome-wide significance cutoff of 0.21 (empirical *p* value < 0.05). Between Asian and African populations, the value is 0.28, slightly lower than the 5 % significance cutoff of 0.34 [39]. The signals of population differentiation of SNP rs9366637 were further confirmed in another data set, the 1000 Genomes Project [40, 41]. The F_ST_ statistics for rs9366637 are 0.35 (empirical *p* value = 0.017) between Asians and Europeans, 0.33 (empirical *p* value = 0.039) between Asians and Africans, and 0.30 (empirical *p* value = 0.026) over the three continental populations. The difference of derived allele frequency (ΔDAF) between Asians and Europeans or Africans are also among the top 5 % highest of genome-wide SNPs (empirical *p* values are 0.016 and 0.024, respectively). The presence of signals of positive selection suggest the functional importance of the Asian-common haplotype. Consistent with our evolutionary analysis, previous phenotypic association studies have shown that the T allele of rs9366637 is associated with increased levels of circulating ferritin in women [42, 43], and also with higher birth weight in newborns [44].

**Fig. 2:**
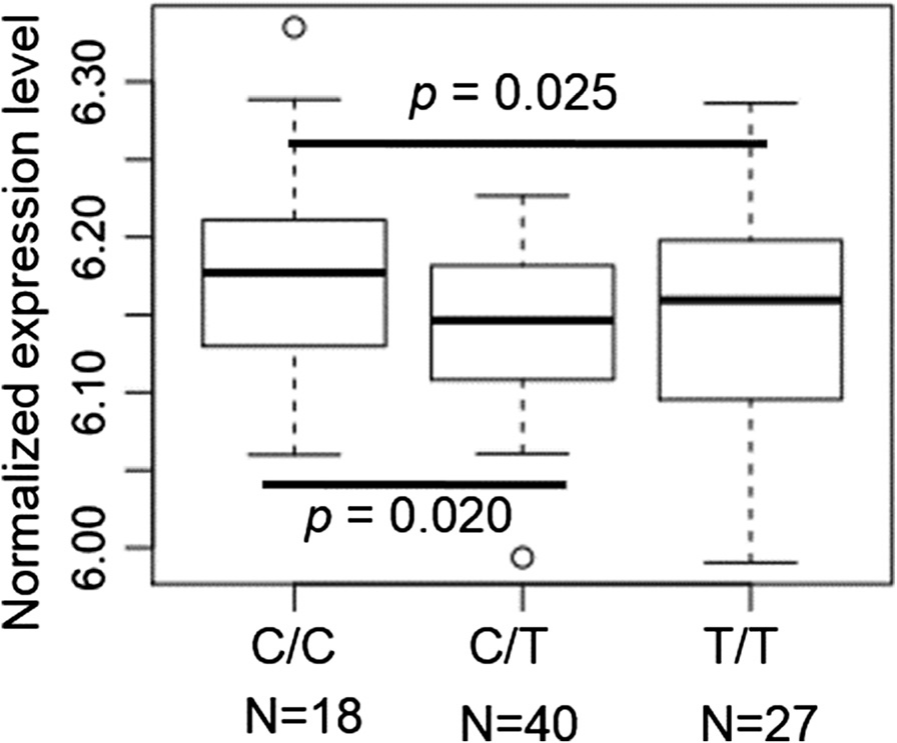
Reduced *HFE* expression in carriers of T at SNP rs9366637. Normalized expression level of *HFE* were measured in the lymphoblastoid cell lines of 43 Chinese individuals from Beijing, China and 42 Japanese individuals from Tokyo, Japan. Individuals with the C/C genotype had significantly higher *HFE* expression when compared to those with the C/T or T/T genotype (Student’s t test, p < 0.05)

The relationship between the genotype of rs9366637 and the *HFE* expression level was assessed using the genotype and gene expression data of Asian individuals in the Hapmap Project. Individuals carrying the genotype C/T or T/T had a significantly lower expression level of *HFE* in lymphoblastoid cell lines (*p* = 0.02, 0.025, respectively, Fig. 2), suggesting that the Asian-common haplotype may carry a regulatory variant(s) that down-regulates the expression of *HFE*. The comparable expression level between C/T and T/T individuals also suggests a dominant effect of the T allele. The lower *HFE* expression of C/T than C/C individuals was further confirmed in four tissues, including lung, tibial nerve, EBV-transformed lymphocytes and prostate (Additional file 1: Figure S3), with data from the Genotype-Tissue Expression program (GTEx) [45]. No splicing effect was observed for rs9366637 on the relative expression of different *HFE* transcripts using 9 tissues from the GTEx Project. As a reduced expression of *HFE* theoretically would lower the expression of hepcidin and consequently lead to enhanced non-heme Fe absorption [9], we further investigated the impact of this Asian-common haplotype on Fe absorption and Fe status.

### Asian women carrying the Asian-common haplotype have higher iron absorption

**Fig. 3:**
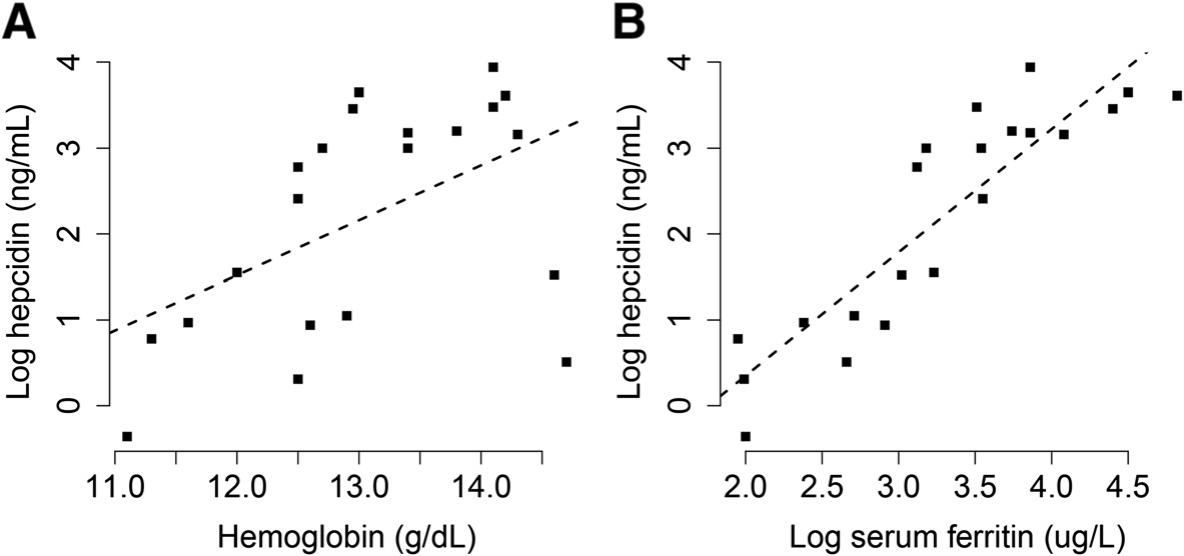
Correlations between hepcidin and iron status markers. Iron status markers include **a**) hemoglobin and **b**) serum ferritin from 21 Asian women participating in the Fe absorption study. Hepcidin and serum ferritin were transformed by natural logarithm. Black dashed lines represent the linear regression line. The correlations between iron status markers and hepcidin were significant: hemoglobin (*p* = 0.018, R^2^ = 0.22), serum ferritin (*p* = 4.24e-08, R^2^ = 0.79). Similar patterns were observed when using data from the screening day

The effect of the Asian-common haplotype on non-heme Fe absorption was assessed in a Fe absorption study involving a group of Asian women volunteers. Only women who were homozygous at the tag SNP, rs9366637, were included in the study so that the participants were either homozygous carriers (with T/T genotype at the tag SNP) or non-carriers (with C/C genotype) of the Asian-common haplotype. Candidate participants were identified through genotype screening on 57 healthy Asian women participants (Methods). In total, 10 non-carriers (8 Chinese, 1 Vietnamese, and 1 Korean) and 11 homozygous carriers (9 Chinese and 2 Korean) of similar age consented to participate in the Fe absorption study. These women participants did not have any health conditions that may cause abnormal Fe status. Specifically, they had normal serum concentrations of folate (>5 ng/mL), vitamin B-12 (>200 pg/mL) and C-reactive protein (CRP) (<10 mg/L), excluding possible influences on Fe status from vitamin deficiency and inflammation [46]. Fe status markers, including hemoglobin (Hb), serum ferritin (SF), serum transferrin receptor (sTfR) and hepcidin, were measured three times on the genotype screening day (Additional file 1: Table S2), on the dosing day when they consumed the Fe tracer (Additional file 1: Table S3), and two weeks after the dosing day (Table 1). All three datasets of Fe status revealed similar patterns presented as follows. Firstly, consistent with the fact that hepcidin is the master regulator of Fe status [9], there were strong associations between hepcidin and Hb (*p* = 0.018, R^2^ = 0.22, Fig. 3a), and SF (*p* = 4.24e-08, R^2^ = 0.79, Fig. 3b). Secondly, compared between the two genotypes, while the concentrations of Hb, SF and hepcidin are similar, sTfR is significantly higher in homozygous carriers than in non-carriers (*p* = 0.021, Table 1).

**Table 1:** Iron status indicators in Asian women participating in the iron absorption study

Variable	C/C (*N* = 10)	T/T (*N* = 11)
Age (y)	22.8 ± 4.0(18-31)	21.2 ± 1.8 (18-24)
Hemoglobin (g/dL)	12.8 ± 0.8 (11.3-14.1)	13.3 ± 1.2 (11.1-14.7)
Serum ferritin (ug/L)	36.3 ± 28.3 (7-89.7)	36.7 ± 33.6 (7.4-124)
Serum transferrin receptor (mg/L)	3.2 ± 1.2 (1.7-5.0)	4.76 ± 1.81* (2.7-8.9)
Serum hepcidin (ng/mL)	17.8 ± 14.2 (1.4-38.5)	16.0 ± 16.7 (0.7-51.5)

NOTE.—Data are expressed as the mean ± SD with the range in parentheses. Iron status indicators were measured in blood obtained 2-weeks post-dosing. Similar patterns were observed using data from the screening day and the dosing day. * indicates *p* = 0.021 in one-tailed Wilcoxon rank sum test between the two genotypes

**Fig. 4:**
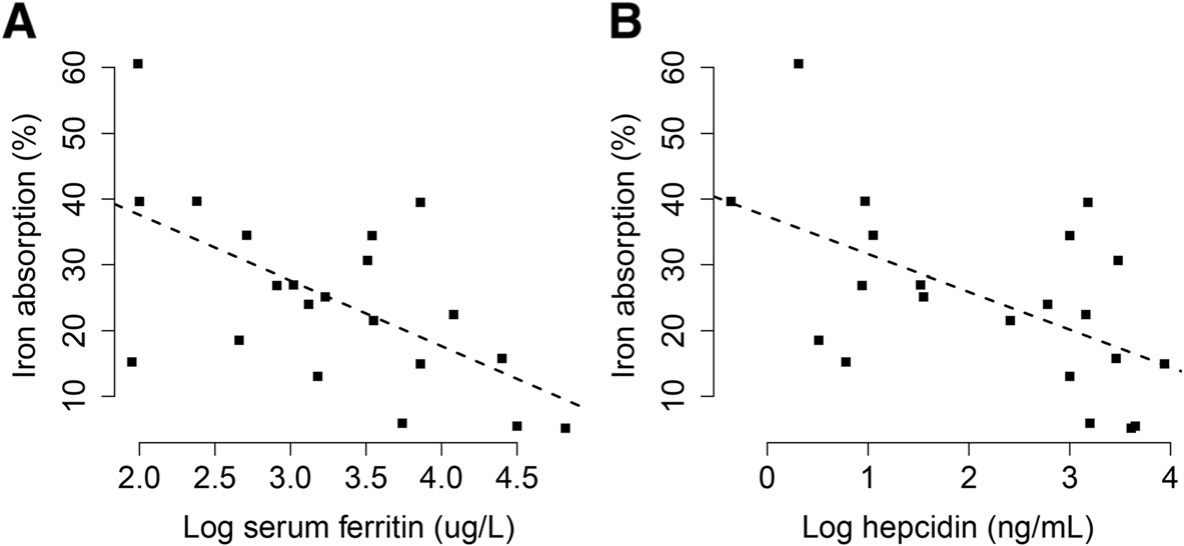
Correlations between iron absorption and iron status markers. Iron status markers included **a**) Serum ferritin and **b**) hepcidin from 21 Asian women participating in the Fe absorption study. Concentrations of serum ferritin and hepcidin were measured in blood obtained 2 weeks after the stable iron isotope was administered. Hepcidin and serum ferritin were transformed by natural logarithm. The black dashed line represents the linear regression line. The correlations were all significant: serum ferritin (*p* = 0.004, R^2^ = 0.33) and hepcidin (*p* = 0.009, R^2^ = 0.27)

**Fig. 5:**
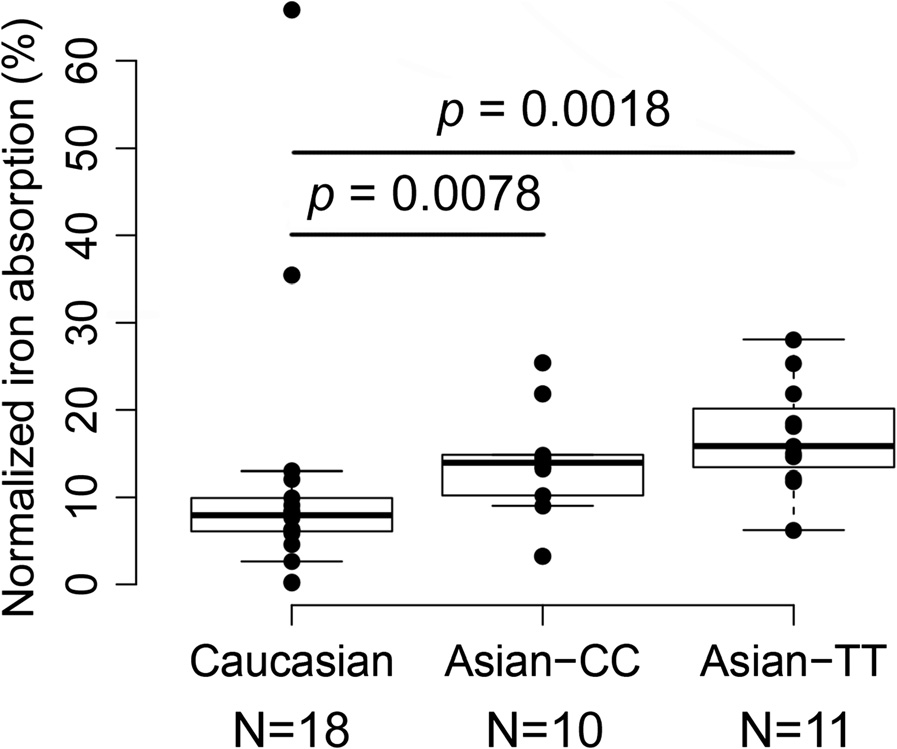
Normalized percent iron absorption in 21 Asian and 18 Caucasian women. Percent iron absorption was normalized to a fixed serum ferritin concentration (40 ug/L). The Asian women were split into two groups based on their genotype at SNP rs9366637. Significantly higher percent iron absorption was evident in Asians of both genotypes

The percent Fe absorption in these 21 women was highly variable (ranging from 5.1 to 60.6 %) and was inversely associated with SF (*p* = 0.004, R^2^ = 0.33, Fig. 4a) and serum hepcidin (*p* = 0.009, R^2^ = 0.27, Fig. 4b). In order to control for the well-known effect of Fe status on intestinal Fe absorption efficiency, Fe absorption data were normalized to a fixed serum ferritin concentration of 40 ug/L [47]. Mean normalized Fe absorption data are presented in Fig. 5. After normalization, elevated percent Fe absorption was evident in homozygous carriers, although the difference only approached significance (*p* = 0.099). The absolute difference in percent Fe absorption between the two genotypes was 3.1 %, which reflected an average increase of 22 % non-heme iron absorption in female homozygous carriers of the Asian-common haplotype when compared with non-carriers in the face of similar Fe stores.

### Asian women have higher iron absorption than Caucasian women

To test if there are population differences in Fe status and absorption between Asians and Caucasians, our data were compared to data obtained from 18 healthy, similarly aged Caucasian women recruited from the same university campus using similar enrollment criteria and investigated with the same experimental approaches [46]. Comparison of characteristics between the groups are presented in Additional file 1: Table S4. No significant Fe status differences were found between the 21 Asian women participating in the Fe absorption study and the 18 age-matched Caucasian women. However, percent Fe absorption normalized to a fixed SF concentration (40 ug/L) was significantly higher in Asian women independent of genotype when compared to the Caucasian cohort (Fig. 5). Caucasian women absorbed 12.02 ± 15.29 % of the supplemental non-heme Fe, while Asian women with C/C genotype absorbed 16 % more (*p* = 0.0078) and those with T/T genotype absorbed 42 % more (*p* = 0.0018). Averaged over the two genotypes, Asian women absorbed 30 % more non-heme Fe than Caucasians (*p* = 0.00053). The independence of elevated non-heme Fe absorption on *HFE* haplotype indicated that there might be other absorption-enhancing genetic variants in Asians.

## Discussion

This study utilized a multidisciplinary approach combining evolutionary analyses and biomedical mass spectrometry to identify genetic variants in *HFE* that may have a functional impact on non-heme Fe absorption in Asian populations. Evolutionary analyses identified an Asian-common haplotype in the *HFE* gene that may have been under positive selection during evolution. Gene expression analyses found significantly lower transcription of *HFE* among carriers of this haplotype. In support of a possible evolutionary advantage, Asian subjects homozygous for this haplotype exhibited higher non-heme Fe absorption after controlling for Fe storage. Additionally, after controlling for Fe status, Asian women exhibited significantly higher non-heme Fe absorption when compared to age-matched Caucasian women, supportive of a population differential genetic basis for Fe homeostasis.

Our evolutionary analyses using genotype data from multiple continental populations confirmed the presence of an Asian-common *HFE* haplotype and positive selection signals on *HFE* in Asian populations [37, 38]. We hypothesized that this frequency shift was due to the selection pressure from the traditional plant-based diet in Asia, which is known to be low in bioavailable Fe [25, 26]. Genetic mutations that enhance the ability to absorb non-heme Fe from a plant-based diet may have increased the ability to maintain adequate Fe status and thus incurred positive natural selection. In support of this hypothesis, the Asian-common-haplotype-tagging allele (T) of the tag SNP rs9366637 is associated with increased levels of circulating ferritin in women [42, 43], and also with higher birth weight in newborns, as were the well-studied *HFE* SNPs, C282Y and H63D [44]. An additional search in GRASP (Genome-Wide Repository of Associations Between SNPs and Phenotypes) [48] with SNPs in high linkage disequilibrium (r^2^ > 0.8) with rs9366637 in Asian populations revealed that one SNP rs7753826 is associated with mean corpuscular hemoglobin concentration (MCHC), Hb, and mean corpuscular volume (MCV) [49, 50], supporting the involvement of rs9366637 and its linked SNPs in Fe homeostasis regulation. Furthermore, individuals carrying the Asian-common haplotype exhibited reduced *HFE* expression. A reduced expression of *HFE* theoretically would lower the expression of hepcidin and consequently lead to enhanced Fe absorption [9]. Although the association between the T allele and lower *HFE* expression was observed consistently in the lymphoblastoid cell lines of Asian individuals from the Hapmap Project (Fig. 2), and also in four tissues from the GTEx Project (Additional file 1: Figure S3), it is noteworthy that this association is not significant in all tissues and cells included in the GTEx project, including liver, where *HFE* mainly functions [51]. It is likely that the small sample size of liver samples (*N* = 31) in the GTEx project and their non-Asian ancestry prevent the detection of the association. In the future, a large number of liver samples from individuals of Asian origin will be ideal to further test this regulatory effect. Additionally, future in-depth analysis on the signatures of positive selection on *HFE* is needed. Although positive selection signals on *HFE* have been consistently observed, the underlying causal variant on the Asian-common haplotype is unclear. The observed associations may be due to a beneficial coding or regulatory variant for *HFE* or even nearby genes. It is noteworthy that *HFE* locates within a cluster of histone genes, which also possess signals of positive selection as detected with the iHS statistic [39]. The possibility could not be ruled out that the *HFE* Asian-common haplotype has hitchhiked the positive selection on nearby histone genes to reach high frequency.

To directly examine the impact of *HFE* haplotypes on Fe absorption, a stable Fe isotope absorption study was undertaken. Young women homozygous for the Asian-common haplotype exhibited elevated non-heme Fe absorption when compared to women homozygous for other haplotypes after controlling for SF concentration. However, the difference observed only approached significance likely due to our limited sample size. Power analysis based on our data suggested that a sample size of 54 individuals per genotype would be required to detect this difference as significant with 80 % power. While the 3 % absolute difference in absorption may not appear substantial, it is actually equivalent to a 14 ~ 20 ug/L decrease in SF concentration based on the relationship between SF and percent Fe absorption observed in this and previous studies [52]. Considering that there are no physiological regulatable routes of Fe excretion and excess Fe accumulation continues across the lifespan [53], a 22 % increase in percent Fe absorption might have significant health implications if maintained across the lifecycle because high iron status has been linked to an increased risk of metabolic diseases [18, 21, 22].

Fe status is one of the major determinants of non-heme Fe absorption, with either SF or serum hepcidin explaining approximately 30 % of the variation in intestinal Fe absorption [46, 54, 55]. High Fe status leads to increased hepcidin expression, which inhibits intestinal Fe absorption and suppresses the release of Fe from body stores [9]. On the other hand, ID or increased erythropoiesis reduces hepcidin expression to allow for enhanced Fe mobilization and utilization [9, 56]. Testing for potential differences in biochemical indicators of Fe status between the two genotypes, only one marker, sTfR, was significantly impacted. The previously reported association between rs9366637 and serum (or plasma) ferritin was not significant in our data set, although consistent with previous observations, SF was elevated for both homozygous and heterozygous carriers of the derived allele, T (Additional file 1: Table S2) [42, 43]. The lack of statistical significance was probably due to our small sample size and the small effect of the SNP on ferritin as reported. In our data set, sTfR was significantly elevated in female homozygous carriers of the Asian-common haplotype. The sTfR is a truncated version of the membrane-bound transferrin receptor (TfR1) and its concentration is directly proportional to the amount of TfR1 on cell membranes, especially those of erythroid precursors [56]. TfR1 binds not only to Fe-loaded transferrin but also to HFE on the membrane of hepatocyte. Since TfR1 has a higher affinity for transferrin, when Fe-loaded transferrin is abundant, HFE is released from TfR1 and binds to transferrin receptor 2 (TfR2). The interaction between HFE and TfR2 is required to up-regulate hepcidin and subsequently down-regulate Fe absorption [29]. Increased TfR1 on the cell membrane, as suggested by elevated sTfR, may prevent HFE from interacting with TfR2 thereby enhancing Fe absorption. Elevated sTfR has been associated with increased Fe absorption in some [56–59], but not all studies [35, 46, 60]. Significant associations between sTfR and serum hepcidin have typically not been reported [46, 60, 61]. In our data, we did not observe any significant correlations between sTfR and Fe absorption or serum hepcidin. The effect of elevated sTfR on non-heme Fe absorption is unclear. Additionally, the observed hepcidin concentration did not significantly differ between the two genotypes, probably due to the large variation and our limited sample size. Larger sample sizes will be needed to identify the underlying mechanisms, including the causal variants, responsible for increased Fe absorption in individuals homozygous for the Asian-common haplotype.

Our population comparison analysis identified significantly higher non-heme Fe absorption in Asian females when compared to age-matched healthy Caucasian females recruited from the same city. To our knowledge, this is the first study comparing iron absorption between Asians and Caucasians. In spite of the concern about batch effect, this approach of pooling data from studies carried out at different points in time is a common practice in stable mineral isotopic studies [62, 63]. In the Fe literature this approach is often further utilized for cross-study comparisons by adjusting iron absorption data to a fixed concentration of storage Fe [47]. A unique property with stable isotopes is that the natural abundance of these isotopes is invariable over time. All measurements of Fe absorption are referenced to natural abundance ratios and are thus comparable across studies. Moreover, all chemicals used were ultrapure and unlikely to contribute variability. However, the possibility of batch effect could not be ruled out and larger studies with both population samples studied at the same time are needed to confirm our observations. Our findings support other published data on population differences in Fe status [12, 27]. Specifically, individuals of African descent tend to have higher Fe status when compared to whites and Hispanics of comparable age and sex [27]. Similarly, the United States Centers for Disease Control and Prevention has set race-specific guidelines that define anemia using a lower Hb concentration in African Americans [64]. Our analysis revealed that Asian women had significantly higher Fe absorption. This enhanced non-heme Fe absorption may be once beneficial under conditions of limited Fe availability, but could be now detrimental in the modern dietary environment. Iron absorption enhancing genetic variants, coupled with Fe-rich diets, may contribute to the increased risk of Fe-related complex diseases, such as type 2 diabetes. Indeed, some iron status parameters (such as hemoglobin or serum ferritin) in Asian individuals in this study (Additional file 1: Table S4) are significantly higher than their Caucasian counterparts in the previous studies. Interestingly, an increased iron level has been linked to a higher disease risk for diabetes [18, 21, 22] and Asian Americans are 30–50 % more likely to suffer from this disorder than their Caucasian counterparts [65]. Future studies are needed to assess the impact of population differential Fe absorption, especially on Fe-related complex disorders. Future studies are also needed to unravel the genetic and molecular basis of population differential Fe absorption. In additional to population specific or differential genetic variants, varying expression of Fe-related genes among populations could be responsible. We performed a preliminary analysis using expression data of HapMap samples from four populations. Interestingly, we observed significantly higher expression of hepcidin and lower expression of ferroportin in Chinese individuals in comparison to Europeans (Additional file 1: Figure S4). However, since these expression data were measured in lymphoblastoid cell lines, it is unclear if they correlate with their levels in more biologically relevant tissues, such as hepcidin in hepatocyte and ferroportin on the basolateral membrane. Also, because there are no Fe absorption data for HapMap individuals, we could not test if these gene expression levels are correlated with Fe absorption. In the future, studies measuring both Fe absorption and gene expression in relevant tissues are required to identify genes that have different expression levels among populations and contribute to population differential Fe absorption.

## Conclusions

Our study confirmed the presence of signatures of positive selection on *HFE* in Asian populations and identified a candidate adaptive haplotype. We further showed that this candidate adaptive haplotype was associated with reduced *HFE* expression. More importantly, by measuring Fe absorption in a group of women of Asian descent, we observed a 22 % higher absorption in homozygous carriers of the adaptive haplotype than non-carriers. Additionally, compared with a group of age-matched Caucasian women, Asian women exhibited significantly elevated Fe absorption. Overall, our study revealed possible parallel adaptation of *HFE* gene in Europeans and Asians with different genetic variants and suggested that regional adaptation of *HFE* may have contributed to elevated Fe absorption in Asians. Our study may help explain the population difference in Fe homeostasis and will assist the future nutritional practice of genome-informed dietary recommendations to avoid Fe overload and Fe-related diseases.

## Methods

### Evolutionary analysis

Haplotype structure analysis was conducted with haplotype data downloaded from Hapmap 3 [66]. SNPs within 20 Kb upstream or downstream of gene *HFE* and with a minimum population frequency of 5 % were used. For SNPs that are in perfect linkage disequilibrium (r^2^ = 1) with each other, only one SNP was randomly chosen to remove redundant information. In total, 10 SNPs were selected (rs9295684, rs6942196, rs2794719, rs9366637, rs2071303, rs1800708, rs1572982, rs17596719, rs6918586, and rs1150658). Haplotype information was available for 170 individuals from Beijing, China and Tokyo, Japan (Coded as CHB + JPT), 85 Chinese in Metropolitan Denver, Colorado (CHD), 117 Utah residents with Northern and Western European ancestry from the CEPH collection (CEU) and 115 Yoruba in Ibadan, Nigeria (YRI). Haplotype network analysis was performed using the median-joining algorithm of Network 4.6.0.0 [67]. The root was inferred assuming that the chimpanzee allelic state at each SNP was ancestral.

Evolutionary analysis on *HFE* was performed through Haplotter [39] and HGDP Selection Browser [68]. Haplotter utilized a selection detection test, iHS, to detect signals of positive selection using HapMap data [39]. HGDP Selection Browser presented the global distribution of SNPs in 53 populations [68].

To further confirm observations from the HapMap and HGDP data, we performed evolutionary analysis on *HFE* using three continentally representative populations from the 1000 Genomes Project [40], by querying the 1000 Genomes Selection Browser 1.0 [41]. The three populations used in analysis are: CHB (Han Chinese in Beijing, China); CEU (Utah residents with Northern and Western European ancestry); and YRI (Yoruba in Ibadan, Nigeria).

### Gene expression analysis

The relationship between the rs9366637 genotype and the *HFE* expression level was assessed using the genotype and gene expression data of Asian individuals in the International Hapmap Project. Genotype data were retrieved from Hapmap 3 [66]. The expression level of *HFE* was retrieved from a previous study, which used a commercial whole-genome expression array (Sentrix Human-6 Expression BeadChip version 1, Illumina) to quantify the transcriptional profiles of Epstein-Barr virus–transformed lymphoblastoid cell lines from individuals genotyped in the HapMap Consortium [69]. Expression signals were transformed to the log scale before normalization. The four technical replicates of an individual were quantile-normalized to have the same distribution. The processed expression value for each gene was further normalized across all studied individuals with median normalization so that the value is comparable across individuals [69]. In total, we have both genotype and *HFE* expression data for 43 Chinese individuals from Beijing, China and 42 Japanese individuals from Tokyo, Japan. The association between rs9366637 and *HFE* expression was further tested with data from the Genotype-Tissue Expression (GTEx) Project, which performs genotype-expression association analysis in a large number of human tissue or cell types [45]. The association analysis was performed on the GTEx Portal (http://www.gtexportal.org/) in all tissues or cells available. The splicing effect of rs9366637 on the relative expression of different *HFE* transcripts was tested with the sQTLseekeR method in 9 tissues (adipose, blood, blood vessel, heart, lung, muscle, nerve, skin and thyroid) from the GTEx Project [70].

### Subjects in genotyping and absorption study

Fifty-seven women of Asian descent were recruited through advertisement on the Cornell University campus in Ithaca, NY from August 2012 to May 2013. Women were eligible for the study if they met the following criteria: 1) non-pregnant and 18–35 years old; 2) of Asian descent with both maternal and paternal grandparents from Asia; 3) not taking any vitamin or mineral supplements for at least 1 month before the study and during the 2-week dosing study interval; 4) without pre-existing medical problems including malabsorption, blood disorders, ulcers, inflammatory diseases, asthma or conditions that might impact inflammation or Fe status; and 5) not taking any prescribed medications known to affect Fe homeostasis. Informed written consent was obtained from each woman, and the study was approved by the Institutional Review Board at Cornell University. A sample size of 60 participants was selected based on the frequency of the tag SNP rs9366637 in Asian populations [66] and this sample size was expected to yield roughly 15 women homozygous for either allele. The sample size of about 15 in each group of homozygous individuals was selected based on previous studies examining genetic effects on Fe absorption [35, 36].

For the genotype and Fe status screening study, Asian women came to the Human Metabolic Research Unit (HMRU) at Cornell University and a venous blood sample (10 mL) was collected to determine *HFE* haplotype and baseline Fe status, including hemoglobin (Hb), serum ferritin (SF), serum transferrin receptor (sTfR), and hepcidin. Women carrying genotypes of C/C or T/T at SNP rs9366637, were then invited to further participate in an iron absorption study, which required two additional visits to the HMRU. On the first day of the absorption study, designated as the dosing day, fasted (≥ 1.5 h) women arrived at the HMRU at which time a baseline weight was obtained and a venous blood sample was collected by either finger stick or venipuncture (< 3 mL) based on subject preference. Participants were then given an oral dose of ^57^Fe tracer as ferrous sulfate (6.3 mg ^57^Fe; total Fe dose of 6.6 mg) flavored with raspberry syrup (Humco, Texarkana, TX). The dose was administered by syringe, which was pre- and post-weighted to calculate the amount of Fe tracer consumed. After consumption, participants remained fasted for an additional 1.5 h, after which they were provided a standardized lunch of vegetable soup, pretzels and water. Two weeks after the ingestion of the Fe tracer, designated as the post-dosing day, participants returned to the HMRU and a 10 mL venous blood samples was collected for analysis of red blood cell ^57^Fe enrichment and serum Fe status indicators.

### Laboratory analysis

DNA was extracted from the whole blood samples of the 57 Asian participants using a commercially available Genomic DNA Purification Kit (Promega Corporation, Fitchburg, WI). A 439 bp long segment centering on rs9366637 was PCR amplified (forward primer: 5′-ATGGTACACTGGGCTTTGGT-3′; reverse primer: 5′-TAGTGCTGAGAAAACCCGCTT-3′) and sequenced by a Sanger sequencing platform (ABI 3730xl). The genotype at rs9366637 (C/C, C/T, T/T) was visually identified from chromatograms using Chromas Lite 2.1.1 software.

Hb and hematocrit were analyzed with a hematology analyzer (Beckman Coulter, Fullerton, CA). SF was measured by a commercially available enzyme immunoassay procedure (Ramco Laboratories Inc, Stafford, TX). The concentration of sTfR was measured with an enzyme-linked immunosorbent assay (ELISA; Ramco Laboratories Inc, Stafford, TX). Serum folate, vitamin B-12, and C-reactive protein (CRP) were measured by Immulite 2000 immunoassay system (Siemens Medical Solutions Diagnostics, Los Angeles, CA). Hepcidin was determined with a commercial Enzyme Immunoassay Kit (S-1337; Bachem, San Carlos, CA).

### Isotope preparation and sample analysis

Fe isotope (^57^Fe at 95 % enrichment) was purchased as the metal from Trace Sciences International (Richmond Hill, Canada). The tracer was converted into a sterile, pyrogen-free solution of ferrous sulfate following the methods used by Kastenmayer et al. [71]. The isotopic composition of the tracer solution was validated with the use of a ThermoQuest Triton TI Magnetic Sector Thermal Ionization Mass Spectrometer (ThermoQuest Corporation, Bremen, Germany).

Whole-blood samples (0.5 mL) were digested with 4 mL Ultrex nitric acid in a polytetrafluoroethylene beaker. Samples were then dried on a hot plate and dissolved in 6N ultrapure hydrochloric acid (JT Baker, Phillipsburg, NJ). Fe was extracted with the use of modified anion exchange chromatography as previously described [46]. Extracted samples were reconstituted in 3 % nitric acid and loaded onto a rhenium filament (H Cross Co, Weehawken, NY) with 4 μL of silica gel (Sigma-Aldrich Inc, St Louis, MO) and 4 μL of phosphoric acid (0.7 N). Isotopic ratio of ^57^Fe to ^56^Fe (^57/56^Fe) was measured by thermal ionization mass spectrometry and compared to the natural abundance of ^57/56^Fe (0.02317). Relative SDs of ^57/56^Fe in analyzed samples averaged 0.04 %.

To control for the known effect of Fe stores on Fe absorption, percent Fe absorption was normalized to each woman’s measured SF concentration as follows: normalized percent absorption = raw percent absorption * SF/40 [47].

### Iron absorption in Caucasian women

Eighteen women of Caucasian descent, aged 18–32 y, were recruited on the same university campus starting in the spring of 2007 with similar enrollment criteria as our recruitment for Asian women. Informed written consent was obtained from each subject, and the study was approved by the Institutional Review Board at Cornell University. Fe status markers and other molecular characteristics were measured with the same experimental approaches as in Asian women except that hepcidin was measured by a competitive ELISA (Intrinsic LifeSciences, La Jolla, CA). Non-heme Fe absorption was assayed from a total Fe dose of 7.6 mg of FeSO_4_ given in 1.5 mL of flavored syrup. The same meals were fed on the dosing day as detailed above. Of the total Fe load administered, 0.9 mg was provided as ^58^Fe. The detailed experimental procedure and subject characteristics have been described previously [46]. Genotyping was not done in these samples but it was estimated that 88.36 % of this cohort (16 individuals) were C/C, 11 % (2 individuals) were C/T, and 0.36 % (0 individual) were T/T based on the known frequency of this haplotype among Caucasians [66].

All data included in our project are available upon request.

### Iron status and absorption data analysis

All statistical analyses were performed with R 3.1.0 [72]. Variables that were not normally distributed (SF, hepcidin) were transformed using a natural logarithm. Potential differences in Fe status and Fe absorption as a function of genotypes were tested with a one-tailed two-sample Wilcoxon rank sum test, which is a non-parametric test without making assumptions about the distribution of variables [73]. Our alternative hypothesis was that homozygous carriers of the Asian-common haplotype would have higher Fe status (as determined by Hb, SF, sTfR and TBI) and higher percent Fe absorption than non-carriers. Possible differences in physical characteristics (age, folate, vitamin B-12, CRP, etc.) among women with different genotypes, and between Asians and Caucasians were tested using the two-tailed, two-sample Wilcoxon rank sum test. Simple linear regression analysis was used to determine possible relationships between Fe status (SF, sTfR, and Hb), percent Fe absorption and serum hepcidin concentrations. Comparisons of Fe status and Fe absorption between Asian and Caucasian women were performed using one-tailed two-sample Wilcoxon rank sum test with the alternative hypothesis that Asians have higher Fe status and normalized Fe absorption.

## Additional file

Additional file 1:
**This file includes additional Figures (S1-S4) and Tables (S1-S4).**

## Abbreviations

CEU: Utah residents with Northern and Western European ancestry
CHB: Han Chinese in Beijing, China
CHD: Chinese in Metropolitan Denver, Colorado
CRP: C-reactive protein
Fe: Iron
GRASP: Genome-Wide Repository of Associations Between SNPs and Phenotypes
GTEx: The Genotype-Tissue Expression Program
Hb: Hemoglobin
HMRU: Human Metabolic Research Unit at Cornell University
HH: Hereditary hemochromatosis
ID: Iron deficiency
JPT: Japanese from Tokyo, Japan
MCHC: Mean corpuscular hemoglobin concentration
MCV: Mean corpuscular volume
SF: Serum ferritin
SNP: Single nucleotide polymorphism
sTfR: Serum transferrin receptor
TfR1: Transferrin receptor 1
TfR2: Transferrin receptor 2
YRI: Yoruba in Ibadan Nigeria
ΔDAF: The difference of derived allele frequency between two populations
