# Supplementary material for: Natural selection on HFE in Asian populations contributes to enhanced non-heme iron absorption
Source: BMC Genet. 2015 Jun 10;16:61. doi: 10.1186/s12863-015-0223-y (PMC4460683; doi:10.1186/s12863-015-0223-y)
Supplement: Additional file 1: — This file includes additional Figures (S1-S4) and Tables (S1-S4). [file 12863_2015_223_MOESM1_ESM.docx]

**Natural Selection on *HFE* in Asian Populations Contributes to Enhanced Non-heme Iron Absorption**

**Kaixiong Ye^1*^, Chang Cao^1*^, Xu Lin^2^, Kimberly O O’Brien^1§^, Zhenglong Gu^1§^**

^1^ Division of Nutritional Sciences, Cornell University, Ithaca, NY, USA

^2^ Key Laboratory of Nutrition and Metabolism, Institute for Nutritional Sciences, Shanghai Institutes for Biological Sciences, Chinese Academy of Sciences and Graduate School of the Chinese Academy of Sciences, Shanghai, China

* These authors contributed equally to this work.

^§^ Corresponding authors: Kimberly O’Brien; Zhenglong Gu

Email addresses for other authors:

Kaixiong Ye: [ky279@cornell.edu](mailto:ky279@cornell.edu)

Chang Cao: [cc748@cornell.edu](mailto:cc748@cornell.edu)

Xu Lin: [xlin@sibs.ac.cn](mailto:xlin@sibs.ac.cn)

Kimberly O’Brien: [koo4@cornell.edu](mailto:koo4@cornell.edu)

Zhenglong Gu: [zg27@cornell.edu](mailto:zg27@cornell.edu)


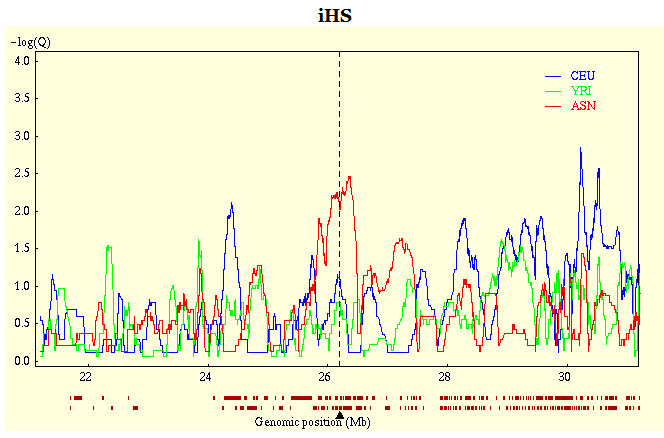


**Figure S1**. Signals of positive selection on *HFE* were evaluated with the iHS method in three continental population samples. This plot was retrieved from the Haplotter database. The x axis represents genomic position with nearby genes indicated as red boxes at the bottom. The target gene, *HFE*, is indicated with a black triangle and a vertical dashed line. The y axis represents the negative logarithm of empirical *p* values for evidence of positive selection. ASN (in red) represents a combination of two samples, coded as CHB and JPT, referring respectively to Han Chinese in Beijing, China and Japanese in Tokyo, Japan. CEU (in blue) refers to Utah residents with Northern and Western European ancestry. YRI (in green) refers to Yoruba in Ibadan, Nigeria.


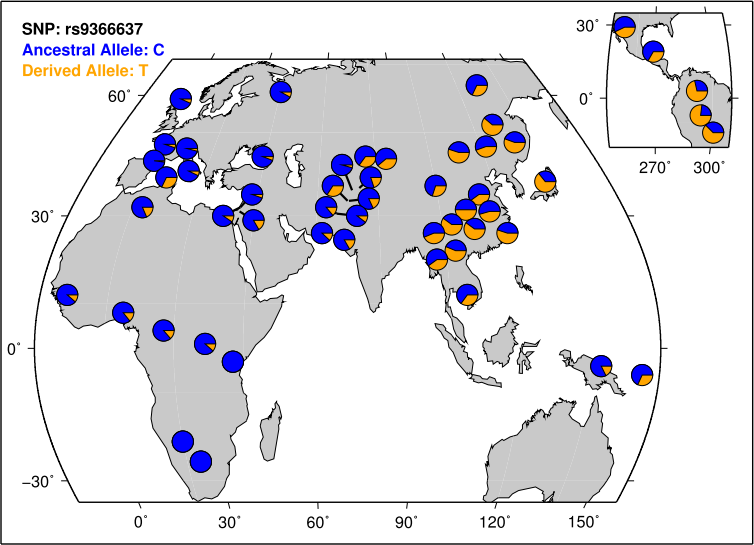


**Figure S2**. The frequency distribution of the tag SNP rs9366637 in global populations. The derived allele T is on the Asian-common haplotype.


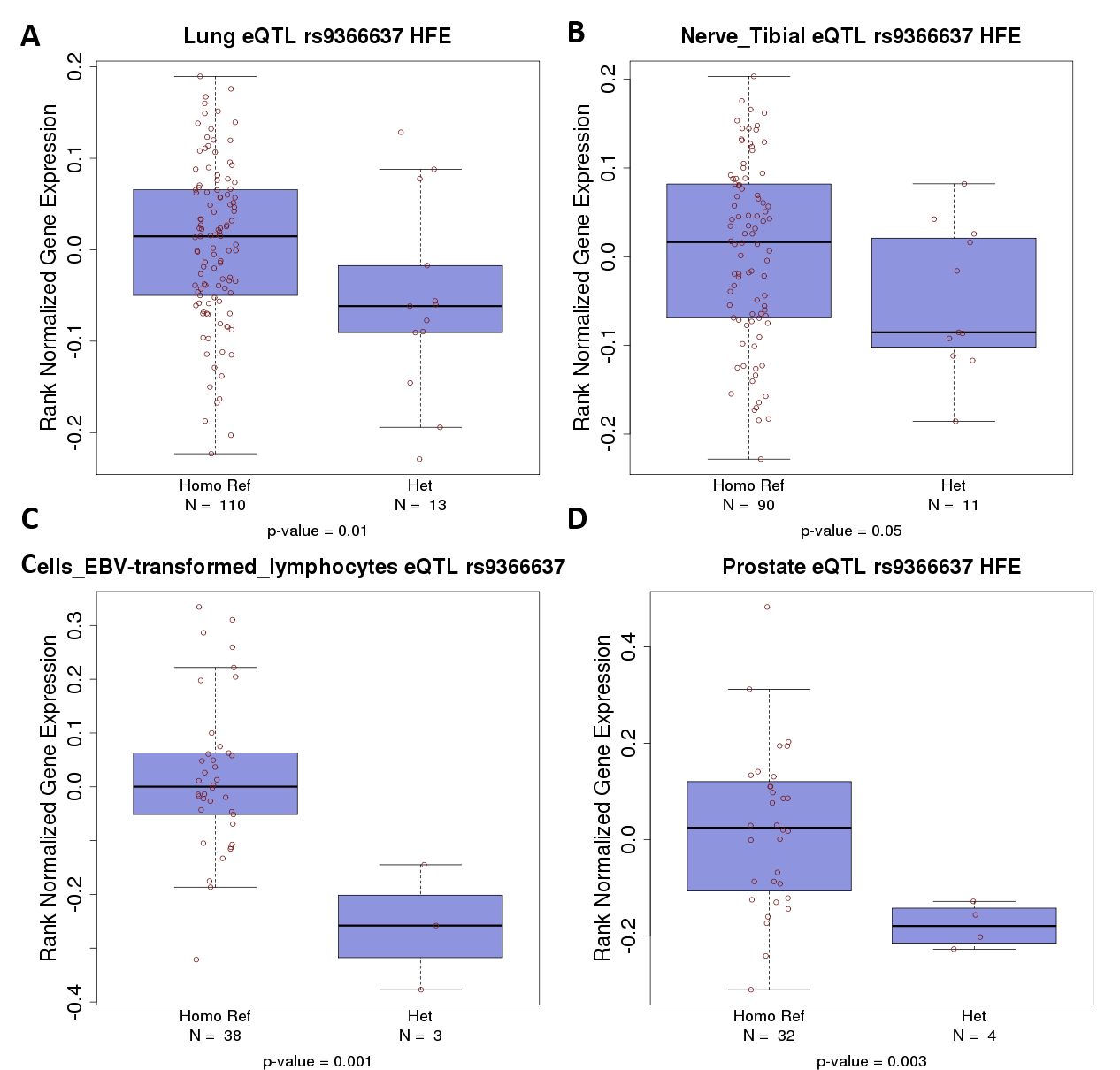


**Figure S3.** Reduced *HFE* expression in heterozygous carriers of the T allele at SNP rs9366637. Significant patterns were detected in four tissues or cells: A) Lung; B) Tibial Nerve; C) EBV-transformed Lymphocytes; and D) Prostate. “Homo Ref” refers to homozygous carriers of the reference allele C at rs9366637. The p value is presented at the bottom of each plot. These plots were retrieved from the GTEx Portal.

**
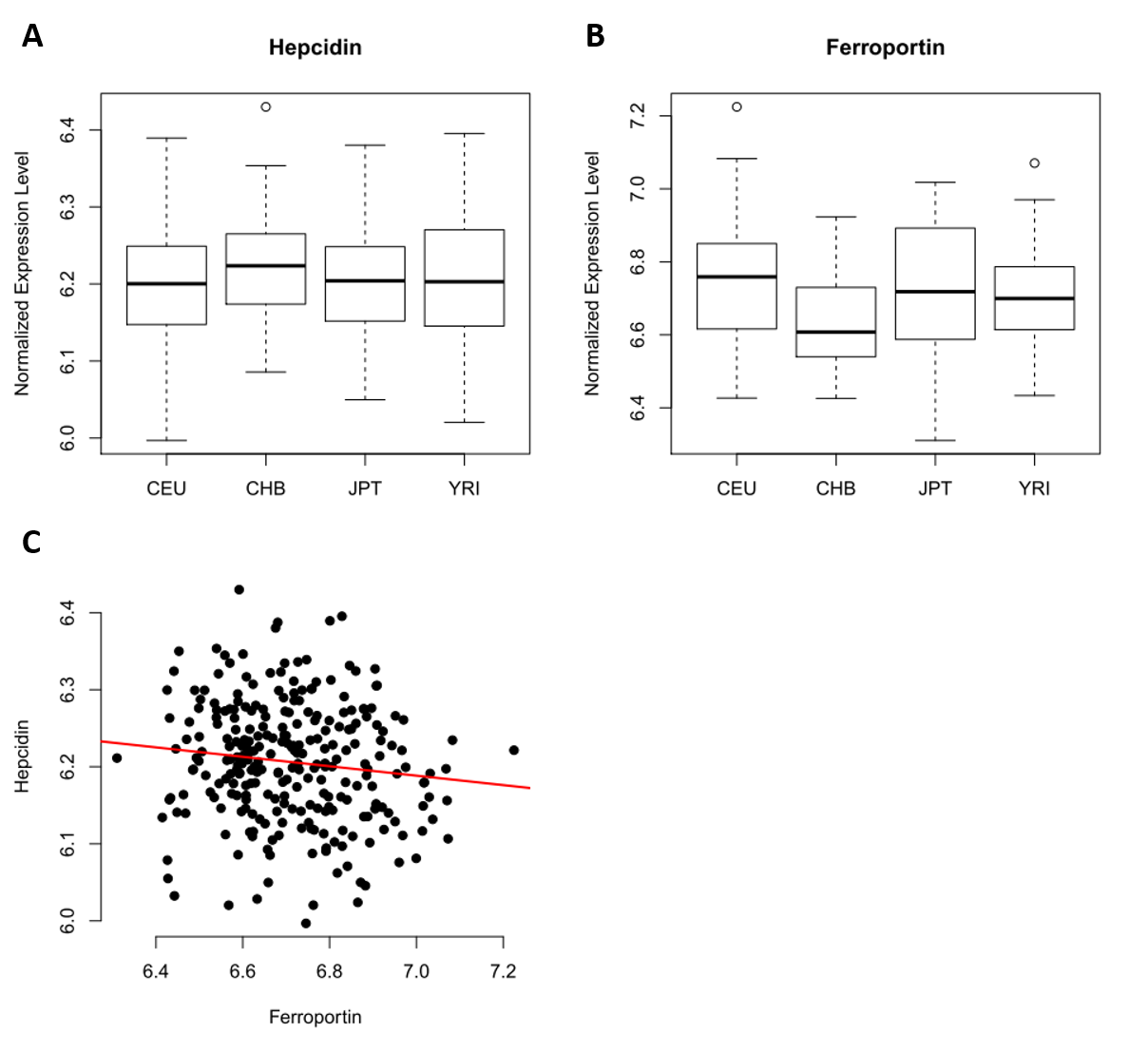
**

**Figure S4.** Expression patterns of hepcidin (*HAMP*) and ferroportin (*SLC40A1*) across populations. Expression data were retrieved from a previous study on HapMap individuals. A) Expression pattern of hepcidin. CHB has significantly higher level than CEU (t test *p* = 0.047). B) Expression patter of ferroportin. CHB has significantly lower level than CEU (*p* = 1.91e-05), JPT (*p* = 0.003) and YRI (*p* = 0.008). C) Negative correlations between the expression level of hepcidin and ferroportin (*p* = 0.042, R^2^=0.01). CHB: Han Chinese in Beijing, China; JPT: Japanese in Tokyo, Japan; CEU: Utah residents with Northern and Western European ancestry; YRI: Yoruba in Ibadan, Nigeria.

**Table S1**. The haplotype of *HFE* in different populations

| **ID** | **Haplotype** | **Total (%)** | **CHB+JPT** | **CHD** | **CEU** | **YRI** |
| --- | --- | --- | --- | --- | --- | --- |
| H1 | CATCTTAGCC | 1 | 0 | 0 | 0 | 0 |
| H2 | TATCCCAGTC | 3(0.31) | 2(0.59) | 1(0.59) | 0(0.00) | 0(0.00) |
| H3 | CGGTTTAACC | 4(0.41) | 2(0.59) | 2(1.18) | 0(0.00) | 0(0.00) |
| H4 | CGGTCCAGCC | 5(0.51) | 0(0.00) | 0(0.00) | 0(0.00) | 5(2.17) |
| H5 | CGGTCTAGCC | 2(0.21) | 0(0.00) | 1(0.59) | 1(0.43) | 0(0.00) |
| H6 | CGGCTTGGTC | 13(1.33) | 2(0.59) | 0(0.00) | 0(0.00) | 11(4.78) |
| H7 | TATCTTGGCT | 16(1.64) | 0(0.00) | 0(0.00) | 0(0.00) | 16(6.96) |
| H8 | CGGCCTAGCC | 40(4.11) | 9(2.65) | 2(1.18) | 28(11.97) | 1(0.43) |
| H9 | TATCTTGGTC | 143(14.68) | 34(10.00) | 27(15.88) | 78(33.33) | 4(1.74) |
| H10 | CATCTTGGTC | 1(0.10) | 1(0.29) | 0(0.00) | 0(0.00) | 0(0.00) |
| H11 | TATCTTGGTT | 39(4.00) | 0(0.00) | 0(0.00) | 1(0.43) | 38(16.52) |
| H12 | TATCCTAGCC | 121(12.42) | 20(5.88) | 4(2.35) | 33(14.10) | 64(27.83) |
| H13 | CGGCTTGGTT | 116(11.91) | 36(10.59) | 27(15.88) | 48(20.51) | 5(2.17) |
| H14 | CGTCTTGGTT | 1(0.10) | 0(0.00) | 0(0.00) | 1(0.43) | 0(0.00) |
| H15 | TAGTCCAGTC | 1(0.10) | 0(0.00) | 1(0.59) | 0(0.00) | 0(0.00) |
| H16 | TATCTTAGCC | 73(7.49) | 0(0.00) | 0(0.00) | 2(0.85) | 71(30.87) |
| H17 | TAGTTTGGTC | 1(0.10) | 1(0.29) | 0(0.00) | 0(0.00) | 0(0.00) |
| H18 | CATCCCAGTC | 38(3.90) | 24(7.06) | 10(5.88) | 4(1.71) | 0(0.00) |
| H19 | CGGTCCAGTC | 299(30.70) | 186(54.71) | 89(52.35) | 14(5.98) | 10(4.35) |
| H20 | CGGCCCAGTC | 1(0.10) | 1(0.29) | 0(0.00) | 0(0.00) | 0(0.00) |
| H21 | CGTCCTAGCC | 1(0.10) | 0(0.00) | 1(0.59) | 0(0.00) | 0(0.00) |
| H22 | CGGCCCAGCC | 4(0.41) | 0(0.00) | 0(0.00) | 0(0.00) | 4(1.74) |
| H23 | TATCTTAACC | 52(5.34) | 22(6.47) | 5(2.94) | 24(10.26) | 1(0.43) |

SNPs included in haplotype analysis are from left to right: rs9295684, rs6942196, rs2794719, rs9366637, rs2071303, rs1800708, rs1572982, rs17596719, rs6918586, rs1150658. Haplotype H1 is the haplotype in Chimpanzees. Haplotype H19, highlighted in blue, is the Asian-common haplotype under study. Haplotypes carrying T at rs9366637 were also highlighted in blue. The column “Total” records the number of a specific haplotype in the overall sample collected from all five populations and the number in the parenthesis is its corresponding percentage among all haplotypes. The last four columns record the number and percentage in each specific sample. CHB: Han Chinese in Beijing, China; JPT: Japanese in Tokyo, Japan; CHD: Chinese in Metropolitan Denver, Colorado; CEU: Utah residents with Northern and Western European ancestry from the CEPH collection; YRI: Yoruba in Ibadan, Nigeria.

**Table S2.** General characteristics and iron status indicators of the 57 study participants as a function of their genotype at rs9366637

| Variable | Total  (N=57) | C/C Genotype  (N=13) | C/C Genotype  (N=10)^a^ | C/T Genotype  (N=33) | T/T Genotype  (N=11) |
| --- | --- | --- | --- | --- | --- |
| Age (y) | 22.7 ± 3.5  (18-34) | 24.0 ± 4.8  (18-34) | 22.8 ± 4.0  (18-31) | 22.7 ± 3.3  (19-31) | 21.2 ± 1.8  (18-24) |
| Folate (ng/mL) | 15.5 ± 4.5  (7.7-28.8) | 16.8 ± 4.6  (9.6-23.9) | 16.6 ± 5.0  (9.6-23.8) | 14.7 ± 4.0  (7.7-23.1) | 16.2 ± 5.7  (8.1-28.8) |
| Vitamin B-12 (pg/mL) | 603.2 ± 255.7  (204.5-1780.5) | 527.3 ± 223.8  (204.5-956) | 519.3 ± 213.1  (298.5-956) | 616.0 ± 188.2 (251-994) | 654.4 ± 426.3  (308.5-1780.5) |
| C-reactive protein (mg/L) | 0.5 ± 0.6  (<0.2-3.0) | 0.8 ± 0.9  (<0.2-3.0) | 0.78 ± 0.97  (<0.2-3.02) | 0.4 ± 0.4  (<0.2-2.4) | 0.6 ± 0.8  (<0.2-2.6) |
| Hemoglobin (g/dL) | 13.3 ± 0.8  (10.9-15.2) | 13.2 ± 0.6  (12.3-14.2) | 13.1 ± 0.6  (12.3-14.2) | 13.4 ± 0.8  (12-15.2) | 13.0 ± 1.0  (10.9-14.4) |
| Serum ferritin (ug/L) | 47.0 ± 35.0  (6.5-183.5) | 38.0 ± 27.7  (10.5-109.1) | 34.8 ± 30.3  (10.5-109.1) | 51.0 ± 36.2  (14.8-183.5) | 45.6 ± 39.7  (6.5-152.8) |
| Serum transferrin receptor (mg/L) | 4.8 ± 2.8  (1.9-15.6) | 4.0 ± 1.7  (1.9-7.5) | 3.8 ± 1.5  (1.9-6.5) | 4.9 ± 3.1  (2.4-15.6) | 5.5 ± 2.8  (2.0-11.7) |
| Serum hepcidin (ng/mL) | 20.1 ± 15.6  (0.6-81.7) | 16.3 ± 11.4  (2.1-36.0) | 13.6 ± 8.6  (2.09-26.6) | 23.1 ± 17.0  (2.5-81.7) | 15.7 ± 14.2  (0.6-46.3) |

NOTE.—Data are expressed as the mean ± SD with the range in parentheses. No significant differences were found among genotypes. The difference of sTfR between C/C and T/T has borderline significance (*p* = 0.06 for all 13 C/C and *p* = 0.07 for 10 C/C participating in the dosing study).

^a^ C/C individuals in the dosing study. Three individuals participating in the screening study did not join the dosing study because they left the city.

**Table S3**. Iron status indicators of study participants on the dosing day

| Variable | C/C | T/T |
| --- | --- | --- |
| Hemoglobin (g/dL) | 14.38 ± 1.93  (12.4-17.4, N=5) | 12.86 ± 1.13  (10.7-14.4, N=8) |
| Serum ferritin (ug/L) | 35.1 ± 27.69  (8.6-97.3, N=10) | 37.27 ± 36.80  (6.3-141.3, N=11) |
| Serum transferrin receptor (mg/L) | 3.36 ± 1.50  (2.01-6.32, N=9) | 5.58 ± 2.76*  (2.48-11.33, N=9) |
| Serum hepcidin (ng/mL) | 12.9 ± 19.6  (1.1-52.3, N=6) | 13.76 ± 9.78  (0.89-26.54, N=8 ) |

NOTE. – Data are expressed as the mean ± SD with the range in parentheses. Because some volunteers donated their baseline blood sample using a finger stick sample instead of a venous blood draw, they did not have enough blood obtained to measure all iron status markers. * indicates *p* = 0.026 in one-tailed Wilcoxon rank sum test between the two genotypes.

**Table S4**. Subject characteristics in Asian and Caucasian samples

| Variable | Caucasian (N=18) | Asian (N=21)^a^ | Asian (N=57)^b^ |
| --- | --- | --- | --- |
| Age (y) | 22.5 ± 3.1  (18-32) | 22.0 ± 3.1  (18-31) | 22.7 ± 3.5  (18-34) |
| Folate (ng/mL) | 18.4 ± 5.5  (11.5-31.9) | 20.3 ± 8.3  (9.2-41.8) | 15.5 ± 4.5  (7.7-28.8) |
| Vitamin B-12 (pg/mL) | 594.6 ± 255.6  (241-1088) | 651.3 ± 373.9  (235.5-1692.5) | 603.2 ± 255.7  (204.5-1780.5) |
| C-reactive protein (mg/L) | 2.9 ± 4.5  (<0.2-15) | 0.31 ± 0.25  (<0.2-0.68) | 0.5 ± 0.6  (<0.2-3.02) |
| Hemoglobin (g/dL) | 12.6 ± 1.3  (11-15.6) | 13.1 ± 1.1  (11.1-14.7) | 13.3 ± 0.8**  (10.9-15.2) |
| Serum ferritin (ug/L) | 38.0 ± 34.1  (5.7-119.7) | 36.5 ± 30.4  (7.0-124.0) | 47.0 ± 35.0*  (6.5-183.5) |
| Serum transferrin receptor (mg/L) | 4.7 ± 1.3  (2.8-7.1) | 4.0 ± 1.7  (1.7-8.9) | 5.4 ± 4.7  (1.9-26.4) |
| Serum hepcidin (ng/mL) | NA | 16.9 ± 15.2  (0.7-51.46) | 20.1 ± 15.6  (0.64-81.72) |

NOTE. – Data are expressed as the mean ± SD with the range in parentheses. * indicates *p* = 0.045, ** indicates *p* = 0.0094 when comparing to the Caucasian data

^a^Data measured on the post-dosing day.

^b^Data measured on the genotype-screening day.
